# Supplementary material for: Identification of photoperiod-induced specific miRNAs in the adrenal glands of Sunite sheep (Ovis aries)
Source: Front Vet Sci. 2022 Jul 22;9:888207. doi: 10.3389/fvets.2022.888207 (PMC9354845; doi:10.3389/fvets.2022.888207)
Supplement: Supplementary file 1 [file Data_Sheet_1.ZIP › Supplementary file/miRDeep2 file.zip!/miRDeep2 file/Know_Structure/SP42A1/oar-let-7g.pdf]

miRBase precursor : oar-let-7g
Total read count : 512886
oar-let-7g read count : 512878
remaining reads : 8

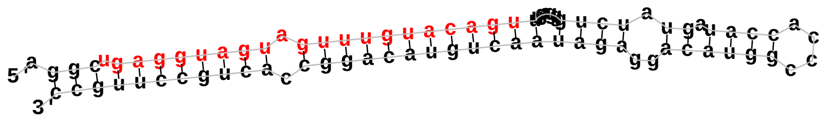

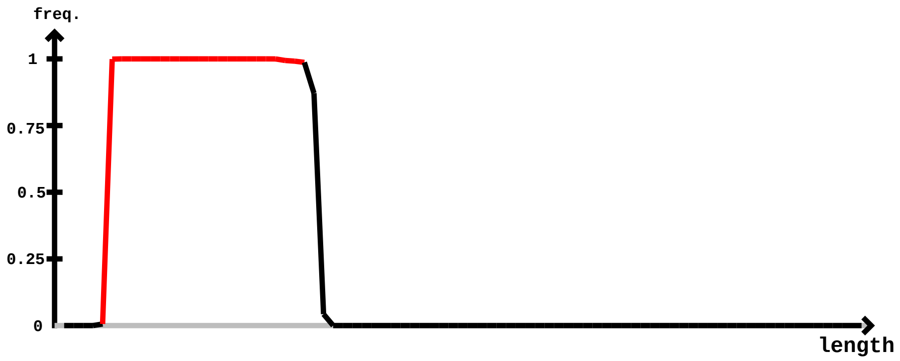

oar-let-7g

5'- aggcugagguaguaguuuguacaguuugagggucuaugauaccacccgguacaggagauaacuguacaggccacugccuugcc -3'	exp		
.(((.((((((((.((((((((((((.....((((.((.((((....))))))..)))))))))))))))).)))))))))))	reads	mm	sample
...cugagguaguaguuuguac...	26	0	seq
...cugagguaguaguuuguaca..	8	0	seq
...cugagguaguaguuuguacag...	20	0	seq
...cugagguaguaguuuguacagu..	531	0	seq
...cugagguaguaguuuguacaguu...	2332	0	seq
...cugagguaguaguuuguacaguuu..	94	0	seq
....ugagguaguaguuugua..	102	0	seq
....ugagguaguaguuugGa..	1	1	seq
....ugUgguaguaguuugua..	1	1	seq
....ugagguagCaguuuguac...	4	1	seq
....ugagguaguaguuuguac...	2952	0	seq
....ugagguaguaguuuguaca..	1307	0	seq
....ugagguaguaguuuguacag...	2277	0	seq
....ugagguaguaguuuguacagu..	58696	0	seq
....ugagguaguaguuuguacaguu...	422307	0	seq
....ugagguaguaguuuguacaguuu..	21809	0	seq
....ugagguaguaguuuguacaguuug...	10	0	seq
....ugagguaguaguuuguacaguuuga..	1	0	seq
....ugagguaguaguuuguacaguuugagggucu..	1	0	seq
.....gagguaguaguuuguaca..	1	0	seq
.....gagguaguaguuuguacag...	2	0	seq
.....gagguaguaguuuguacagu..	43	0	seq
.....gagguaguaguuuguacaguu...	333	0	seq
.....gagguaguaguuuguacaguuu..	16	0	seq
......agguaguaguuuguacaguu...	5	0	seq
....aacuguacaggccacugccuugc.	1	0	seq
....acuguacaggccacugccuugc.	1	0	seq
....cuguacaggccacugccuugc.	2	0	seq
....cuguacaggccacugccuugcc	3	0	seq
